# Controlling the Anionic Ratio and Gradient in Kesterite Technology

**DOI:** 10.1021/acsami.1c21507

**Published:** 2022-01-03

**Authors:** Jacob Andrade-Arvizu, Robert Fonoll Rubio, Victor Izquierdo-Roca, Ignacio Becerril-Romero, Diouldé Sylla, Pedro Vidal-Fuentes, Zacharie Jehl Li-Kao, Angélica Thomere, Sergio Giraldo, Kunal Tiwari, Shahaboddin Resalati, Maxim Guc, Marcel Placidi

**Affiliations:** † Solar Energy Materials and Systems (SEMS), Institut de Recerca en Energia de Catalunya (IREC), Jardins de les Dones de Negre 1, Sant Adrià de Besòs, Barcelona 08930, Spain; ‡ Departament d’Enginyeria Electrònica, Universitat Politècnica de Catalunya, C/ Jordi Girona 1, Barcelona 08034, Spain; § Architectural Engineering Research Group, Oxford Brookes University, Headington Rd, Headington, Oxford OX3 0BP, United Kingdom

**Keywords:** kesterite, Cu_2_ZnSn(S,Se)_4_, Cu_2_ZnGe(S,Se)_4_, anionic control, band gap grading

## Abstract

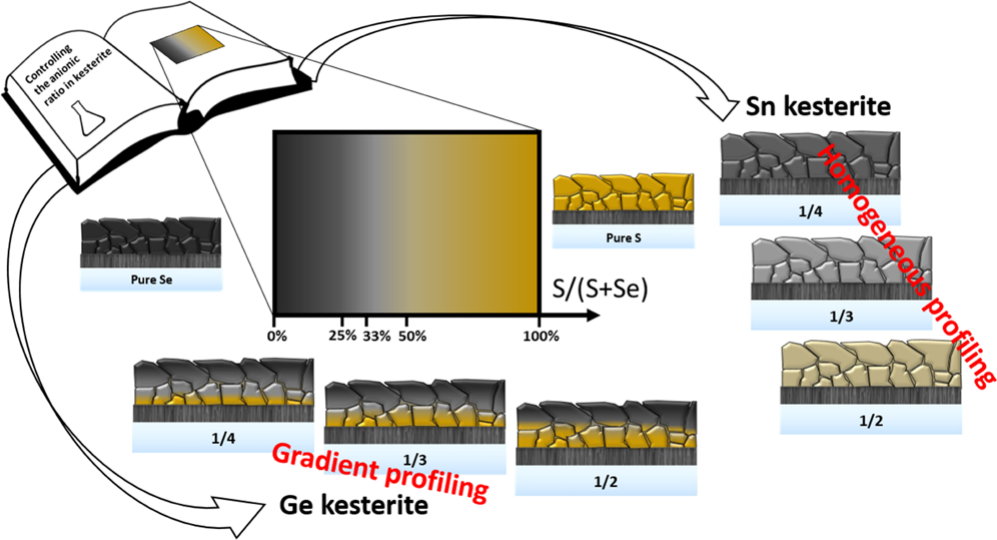

Accurate anionic control during the formation of chalcogenide solid solutions is fundamental for tuning the physicochemical properties of this class of materials. Compositional grading is the key aspect of band gap engineering and is especially valuable at the device interfaces for an optimum band alignment, for controlling interface defects and recombination and for optimizing the formation of carrier-selective contacts. However, a simple and reliable technique that allows standardizing anionic compositional profiles is currently missing for kesterites and the feasibility of achieving a compositional gradient remains a challenging task. This work aims at addressing these issues by a simple and innovative technique. It basically consists of first preparing a pure sulfide absorber with a specific thickness followed by the synthesis of a pure selenide part of complementary thickness on top of it. Specifically, the technique is applied to the synthesis of Cu_2_ZnSn(S,Se)_4_ and Cu_2_ZnGe(S,Se)_4_ kesterite absorbers, and a series of characterizations are performed to understand the anionic redistribution within the absorbers. For identical processing conditions, different Se incorporation dynamics is identified for Sn- and Ge-based kesterites, leading to a homogeneous or graded composition in depth. It is first demonstrated that for Sn-based kesterite the anionic composition can be perfectly controlled through the thicknesses ratio of the sulfide and selenide absorber parts. Then, it is demonstrated that for Ge-based kesterite an anionic (Se–S) gradient is obtained and that by adjusting the processing conditions the composition at the back side can be finely tuned. This technique represents an innovative approach that will help to improve the compositional reproducibility and determine a band gap grading strategy pathway for kesterites. Furthermore, due to its simplicity and reliability, the proposed methodology could be extended to other chalcogenide materials.

## 1 Introduction

Thin-film techniques based on different chalcogenide materials are widely explored owing to their versatility, tailorable properties, and relatively low-cost manufacturing processes, offering a high potential for optoelectronic device application. Frequently, accurate anionic and/or cationic control for the formation of chalcogenide solid solutions is fundamental to the tuning of the physicochemical properties of the compounds and is one of the main issues in the field. A kesterite Cu_2_ZnSn(S,Se)_4_ (CZTSSe)-based thin film is one of the chalcogenide family members that have been more intensively investigated in the last decade owing to its potential to make flexible, light-weight, and low-cost photovoltaic (PV) devices based on earth-abundant materials.^1^ Several groups have demonstrated efficiencies higher than 10%,^2−7^ often with different compositional ratios. However, no consensus exists on reliable processes and reproducible efficiencies, which remain extremely challenging despite ongoing efforts at the different levels of the device. This relates not only to the difficult control of the synthesis process of the material but also to the various layers and interfaces involved in the full solar cell devices. Interfaces within the absorber itself, i.e., defects at the grain boundaries or even in-grain defects, can seriously alter the performance of the devices.^8−10^

Defect formation can be restrained to some extent through fine tuning of the compositional anionic ratio and/or cationic ratio. However, achieving such accurate control is not straightforward, especially if the anions are introduced during the thermal synthesis of the absorbers. A particularly relevant application of precise compositional mastery is the achievement of a graded composition in the absorber. Compositional grading is the key aspect of band engineering and is especially valuable at the device interfaces for an optimum band alignment while also finding application in the control of interface defects and recombination; it is additionally necessary for the optimization of carrier-selective contacts.^11−19^ The preparation of solid solution kesterite phases typically involves mainly either (1) a chemical or physical route already containing a precursor with sulfur or selenium (often in the form of Zn or Sn chalcogenides), which is submitted to a reactive annealing process under an atmosphere containing an alternate chalcogen (i.e., sulfur for selenide precursors and selenium for sulfide precursors),^20,21^ or (2) a direct route with a reactive annealing atmosphere containing both chalcogens.^20−22^ However, in all of these cases, crystallizing the desired compositional phase in a repeatable way is a laborious task and, often, requires tens of processes to properly adjust the S/Se weight ratio of the reaction.

Moreover, the high volatility of S and Se species during the thermal processes renders reproducible control on the ratio of these elements difficult to achieve.^23^ In the same way, and while few reports exist on kesterite absorbers with compositional anionic gradients, similar strategies to the ones reported above were implemented, i.e., including S and Se mixtures during thermal annealing.

Sequential S and Se thermal treatments were used, with sulfide or selenide binaries in the precursor material, and then submitted to reactive annealing introducing the alternate chalcogen, resulting in complicated setups.^12,20^ The straightforward formation of a compositional gradient in a foolproof way remains a challenging task that this work aims to address.

The difficulty of controlling the anionic composition in kesterite compounds is mainly related to the small difference in the formation enthalpies of the end members of the solid solutions (e.g., the difference between Cu_2_ZnSnS_4_/Cu_2_ZnSnSe_4_ or Cu_2_ZnGeS_4_/Cu_2_ZnGeSe_4_ is 1.08 and 0.68 eV, respectively).^24^

As such, directing the S–Se behavior becomes challenging and necessarily implies complex multistep processes rather than a single step in which control is almost impossible to achieve.

In this context, developing a methodology that allows controlling the anionic composition and/or creating anionic compositional grading in a simple, precise, and reproducible way represents a step forward in the development of kesterite-based photovoltaic (PV) technology.

In this work, an innovative approach is proposed for accurately controlling the anionic (sulfur–selenium) compositional ratio in kesterite absorbers that consists of first preparing a pure sulfide absorber with a specific thickness followed by the synthesis of a pure selenide part of complementary thickness on top of it.

We employ this methodology for the synthesis of Cu_2_ZnSn(S,Se)_4_ (CZTSSe) and Cu_2_ZnGe(S,Se)_4_ (CZGSSe) kesterite absorbers, and, through the use of several characterization techniques, we show that Se presents a different incorporation dynamics in each of them, leading to a homogeneous or graded in-depth composition. In this way, we demonstrate that for Sn-based kesterite, the anionic composition can be perfectly regulated through the thicknesses ratio of the sulfide and selenide absorber parts and that for the Ge-based kesterite an anionic (Se–S) gradient is obtained and can be finely tuned by adjusting the processing conditions. While slightly beyond the scope of this work, the leading hypothesis explaining the Se–S dynamics is discussed at the end of this work in regard to the literature.

## 2 Experimental Section

### 2.1 Absorber Synthesis and Device Fabrication

**Figure 1:**
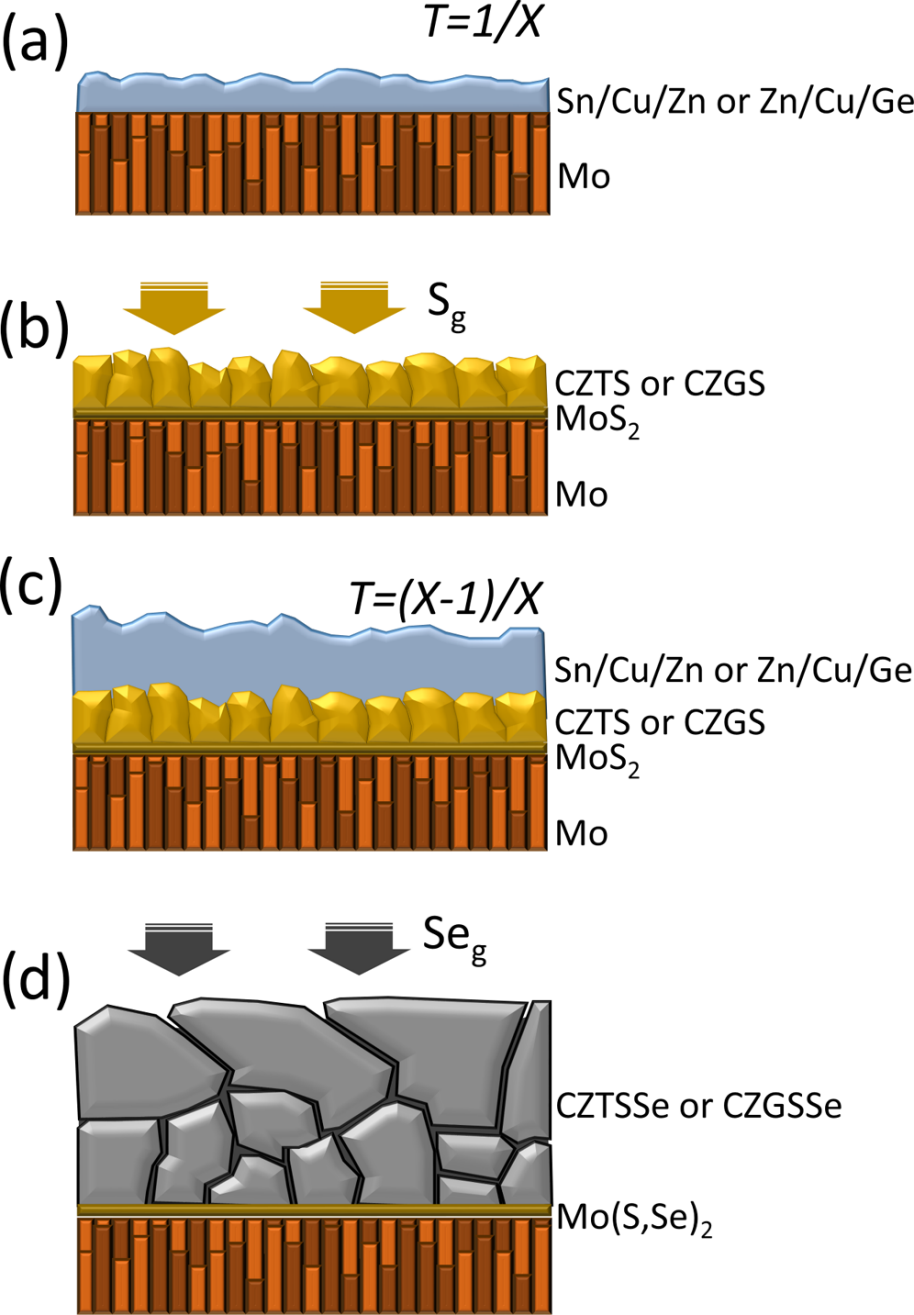
Followed strategy for the preparation of the solid solution samples of the study. (a) Deposition of 1/*X* of the total metallic stack thickness. (b) Sulfurization of the first metallic stack. (c) Second deposition of (*X* – 1)/*X* of the total metallic stack. (d) Final selenization of all of the layers.

The synthesis process began with the deposition (Alliance AC450 DC-sputtering system) of the first metallic precursor with a thickness 1/*X* of the total one (see Figure 1a) on a Mo layer on soda-lime glass. Cu/Sn/Cu/Zn and Cu/Zn/Cu/Ge structures were used for the Sn- and Ge-based kesterite, respectively.^7,25^ In all of the cases, the metallic precursors were adjusted to give Cu-poor and Zn-rich composition ratios ([Cu]/([Zn] + [Sn]) ∼ 0.77 and [Zn]/[Sn] ∼ 1.15 for Sn precursors and [Cu]/([Zn] + [Ge]) ∼ 0.67 and [Zn]/[Ge] ∼ 1.07 for Ge precursors), as measured by X-ray fluorescence (XRF).

The first precursor is submitted to sulfurization (see Figure 1b) in a tubular furnace (Hobersal) with a semiclosed graphite box containing 50 mg of sulfur and using a thermal profile consisting of the first step at 200 °C during 5 min (under 1 mbar Ar flow) and the second step at 550 °C during 5 min (at 1 bar). Then, the second metallic stack precursor was deposited on the sulfide absorber (Figure 1c) with a thickness of (*X* – 1)/*X* of the total one, and the samples were selenized in a tubular furnace with a graphite box containing 50 mg of selenium and using a thermal profile consisting of the first step at 400 °C during 30 min (under 1 mbar Ar flow) and the second step at 550 °C during 15 min (at 1 bar), forming the solid solution (CZTSSe or CZGSSe) absorber (Figure 1d). In the case of Ge kesterite, the second batch of samples was prepared using a lower selenization temperature process, defined by a thermal profile consisting of the first step at 330 °C during 30 min (under 1 mbar Ar flow) and the second step at 480 °C during 15 min (at 1 bar). In all of the absorbers, the thicknesses of the bottom and top metallic precursors were adjusted to have a total absorber thickness of around 1.5 μm. Prior to the thermal processes, the employed graphite boxes were cleaned and submitted to high-temperature (750 °C) annealing in vacuum for 2 h to remove any possible contaminations.

Devices were finally fabricated for both Sn- and Ge-based kesterite by depositing an n-type CdS buffer layer (50 nm) by chemical bath deposition at 80 °C and a window layer formed by an i-ZnO (50 nm) and an indium tin oxide (ITO) (150 nm) layer deposited by DC-pulsed magnetron sputtering (Alliance Concept CT100) at 200 °C. Individual cells (3 × 3 mm^2^) were then insulated using a mechanical scriber (OEG MR200). In both cases, the experimental results are compared and interpreted with the help of a numerical model.

### 2.2 Characterization

Raman spectroscopy measurements were performed using FHR-640 and iHR320 Horiba-Jobin Yvon spectrometers coupled to Raman probes developed at IREC and charge-coupled device (CCD) detectors. A lift-off procedure allowed to reveal the back side of the absorber and the substrate side, and Raman measurements were thus performed at different interfaces. Lift-off was performed by bonding the samples from the front side to a steel substrate with an adhesive epoxy and then removing them mechanically. The back contact–absorber interface in thin-film chalcogenides is generally highly stressed, and the presence of a Mo chalcogenide layer there (formed during the absorber synthesis), which has a layered structure, renders this interface very weak, easing separation. The Raman measurements were performed in backscattering configuration, and different excitation wavelengths (532 and 785 nm) were employed. Due to the difference in the band gap of the different analyzed layers, the penetration depth of the used excitation wavelength was different, enabling an in-depth compositional analysis of the very top surface (with 532 nm excitation) and subsurface (with 785 nm excitation) regions of the absorbers at front and back faces, as well as of the top surface of the substrate. The analysis also included the detection of possible secondary phases at these interfaces. To inhibit thermal effects on the samples, the excitation power density was about 100–150 W/cm^2^. A calibration was performed using a monocrystalline Si reference to correct the Raman shift to the main Si band at 520 cm^–1^. The in-depth chemical composition of the CZTSe absorbers was investigated by means of Auger electron spectroscopy (AES) using a Phi 670 scanning Auger nanoprobe. Cross-sectional scanning electron microscopy (SEM) images were obtained with a ZEISS Series Auriga microscope applying 5 kV as the accelerating voltage and at working distances of 3–5 mm to study the morphology of the absorbers.

The crystallographic features of the CZTSe absorbers were characterized by X-ray diffraction (XRD) using a Bruker D8 Advance system with Cu Kα radiation. The overall composition of the absorber layers was measured with an X-ray fluorescence (XRF) system (FISCHERSCOPE XVD) calibrated by inductively coupled plasma mass spectrometry (ICP-MS).

Current–voltage measurements were performed under AM1.5G illumination (1000 W/m^2^) using a solar simulator (Abet Technologies Sun 3000 Class AAA) at room temperature and calibrated with a Si reference solar cell.

## 3 Characterization Results

### 3.1 Sn Kesterite

**Figure 2:**
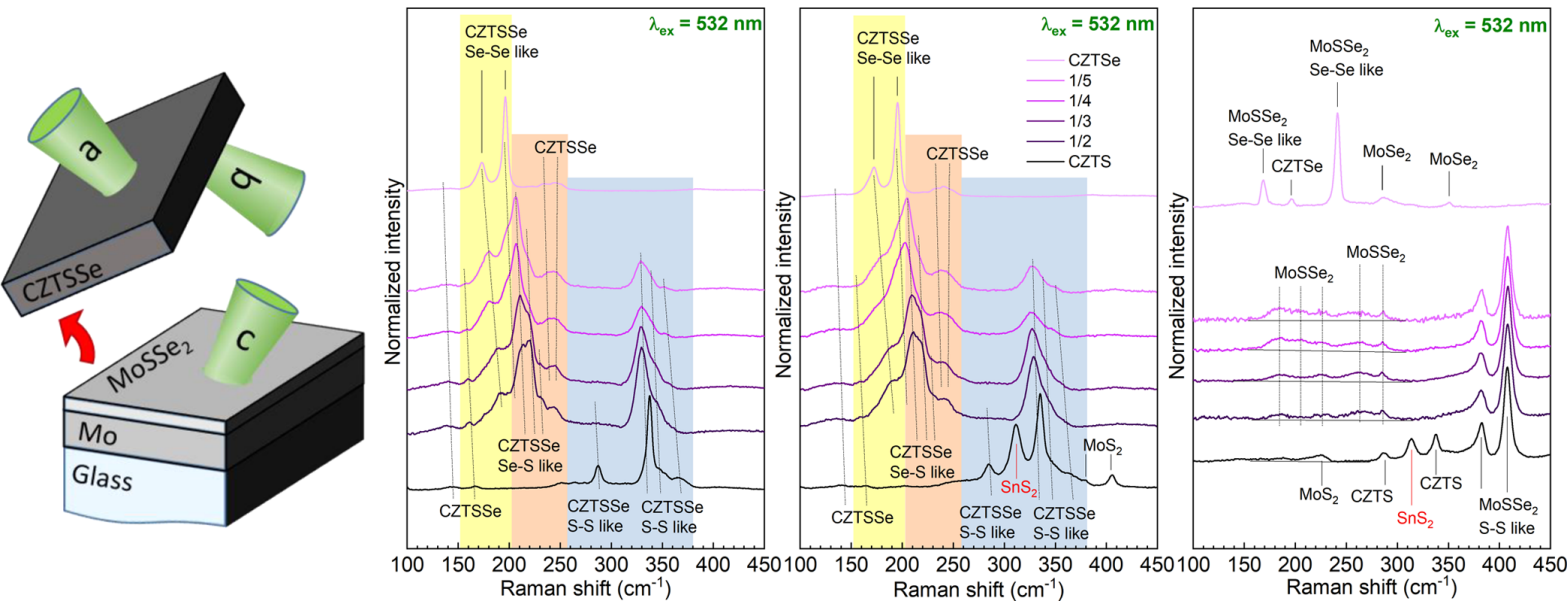
Lift-off procedure scheme (left) revealing the different a, b, and c measured interfaces corresponding to the front surface region, the CZTSSe back contact region, and the Mo back contact region. Raman spectra of (a) front surface, (b) back, and (c) substrate side interfaces of the CZTSSe and pure CZTS and CZTSe compounds measured under 532 nm excitation wavelength.

Raman analysis was performed for all of the CZTSSe absorbers, which allowed us to estimate the anionic composition of the surface. Pure CZTSe has its main Raman peak at 196 cm^–1^ (A symmetry mode), a weaker contribution at 174 cm^–1^ (overlap of the A and B symmetry modes), and a broad band in the 220–250 cm^–1^ region (overlapping of E and B symmetry modes).^26^ Pure CZTS has a similar fingerprint but with well-defined contributions at 337 cm^–1^ (main peak) and 287 cm^–1^ (weaker one) for the A mode and at 310–380 cm^–1^ for E and B modes.^27^ For both pure compounds, the A symmetry modes are related to the pure anion vibrations, while E and B symmetry modes include different combinations of vibrations of cations and anions.^28^ In the case of the CZTSSe solid solution, a more complex situation is observed with three different spectral regions wherein no S (between 150 and 200 cm^–1^) and no Se (250–380 cm^–1^) vibrational modes are present and wherein a combination of vibrations of both anions (200–250 cm^–1^) can be seen.^29^ Depending on the intensity ratio of the different spectral regions, the anionic composition of the CZTSSe solid solution layer can be estimated.^12,30^ In the first step, a multiwavelength (using laser excitations at 532–785 nm) Raman analysis was performed on the front surface (see Figures 2 and S1), taking advantage of the different penetration laser depths. This preliminary result indicated that the anionic composition [S]/([S] + [Se]) of the surface was varying from sample to sample (from approximatively 45–20%) but showing similar values for the surface (using 532 nm) and subsurface (using 785 nm) of each absorber. This anionic composition of the front surface was clearly indicating a mixture of anions in the final CZTSSe absorbers, and it was then decided to perform a complete structural and compositional characterization to detect the presence of an anionic gradient at the back side and/or eventual secondary phases. To complete Raman analysis, the measurements were performed at the back sides of the absorbers and at the Mo back contact side (revealed by a mechanical lift-off procedure) (see Figure 2). Reference spectra of pure CZTSe and CZTS were also added, allowing a better distinguishing of the solid solution compounds. The calculated values for the anionic compositions for the different CZTSSe absorbers are reported in Table 1 and were found to be almost similar for the front and back sides of the absorbers, with only minor changes in some cases, which indicates a homogeneous composition within the thickness of the absorber. Efficient penetration of Se through the whole layer was also confirmed by the formation of the Mo(S,Se)_2_ phase found at the substrate side of the samples. Moreover, a clear change in the anion ratio of the Mo(S,Se)_2_ phase can be deduced from the measured Raman spectra (see Figure 2c), which follows the change in the relative thickness of S and Se precursors.

**Table 1:** Anion Composition [S]/([S] + [Se]) Calculated from Different Techniques, XRF and XRD, Applying Vegard’s Law, Raman Spectra Obtained at the Front and Back of the Samples,^30^ and from Auger Measurements

	XRF				XRD		Raman		AES		
	bulk				bulk		surface (<100 nm)	back (<100 nm)	surface (<100 nm)	bulk	back (<200 nm)^b^
sample	[Cu]/M^a^	[Cu]/[Sn]	[Zn]/[Sn]	[S]/([S] + [Se]) [%]	[S]/([S] + [Se]) [%]	(112) FWHM [°]	[S]/([S] + [Se]) [%]	[S]/([S] + [Se]) [%]	[S]/([S] + [Se]) [%]	[S]/([S] + [Se]) [%]	[S]/([S] + [Se]) [%]
	(±0.3)	(±0.04)	(±0.03)	(±3)	(±1)	(±0.01)	(±3)	(±3)	(±2)	(±2)	(±4)
CZTSe	44.5	1.72	1.14	0	0	0.05	0	0			
1/5	44.7	1.66	1.05	23	21	0.11	18	16	18	19	23
1/4	45.0	1.71	1.09	23	23	0.11	20	17	21	21	25
1/3	45.0	1.72	1.11	36	36	0.11	34	33	36	35	38
1/2	44.4	1.70	1.13	44	42	0.12	43	35	40	41	45
CZTS	44.7	1.69	1.09	100	100	0.07	100	100			

a M = [Cu] + [Zn] + [Sn].

b Sulfur overestimation due to the overlapping of the S and Mo AES signals and the contribution of the S-rich MoSSe_2_ layer.

**Figure 3:**
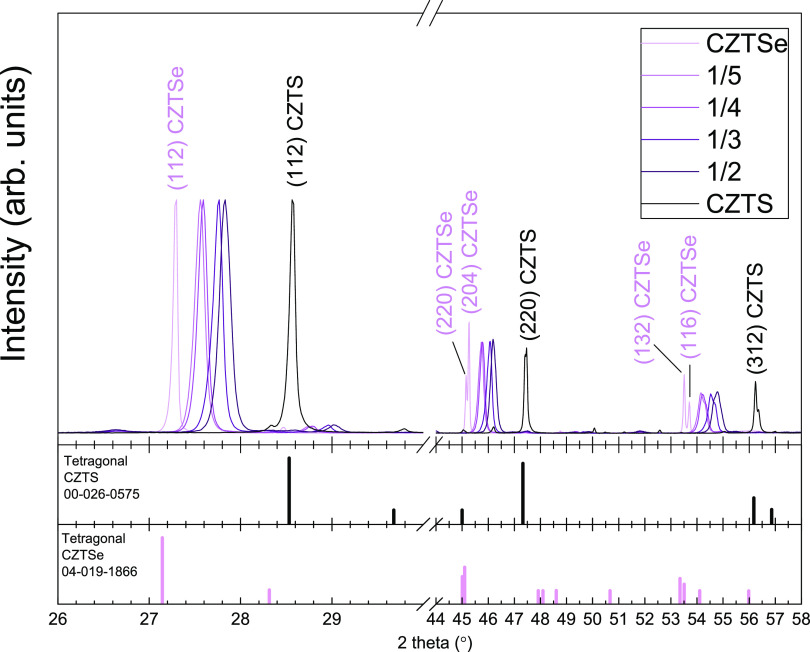
Difractograms of the CZTSSe absorbers and the CZTS and CZTSe reference samples.

**Figure 4:**
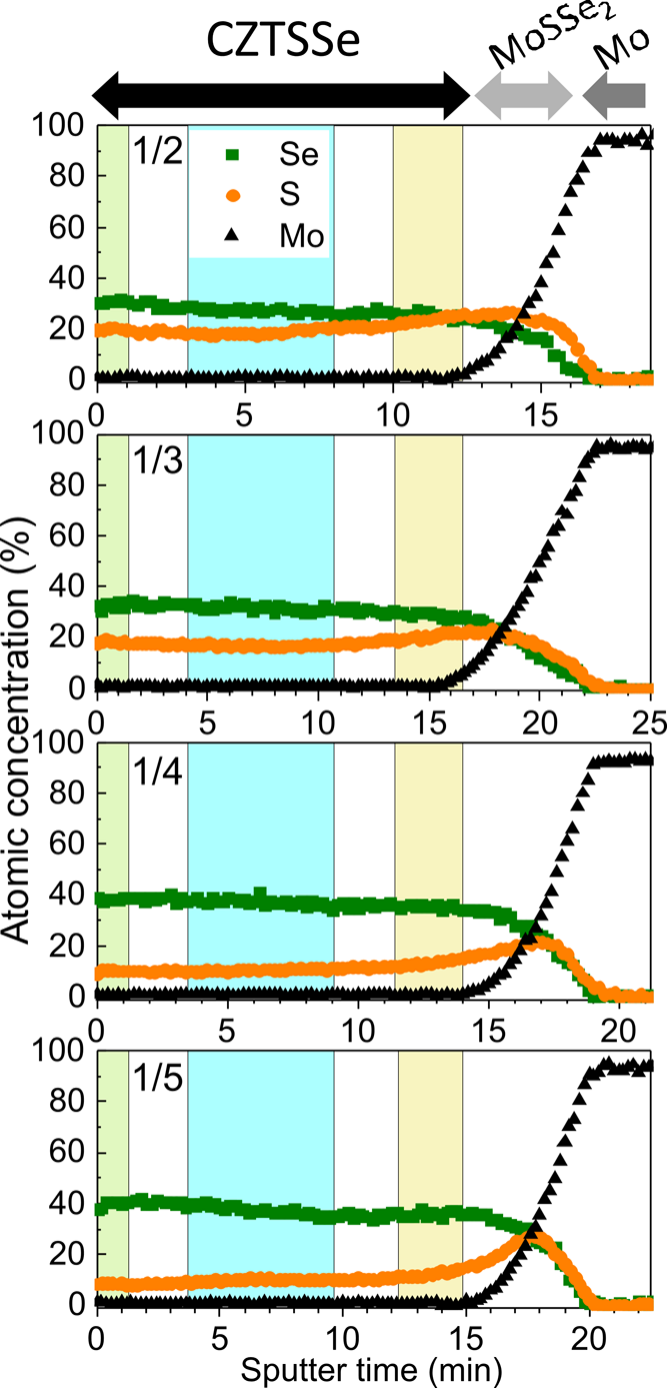
Auger spectroscopy (AES) depth profiles of the CZTSSe samples. The colored region indicates the sample depth used for the evaluation of the composition of the surface (green), bulk (blue), and back (yellow).

To confirm the homogeneous in-depth distribution of the anions in the CZTSSe layers, X-ray diffraction (XRD) measurements and depth-resolved AES analysis were performed. The measured diffractograms, shown in Figure 3, clearly revealed the CZTSSe structure in all of the absorbers, without the presence of separated CZTSe and CZTS phases and also without any evidence of secondary phases, suggesting the formation of the CZTSSe solid solution phase with good anionic compositional homogeneity through all of the thickness of the film without any anionic gradient. Moreover, the systematic shift of the Bragg reflections toward higher diffraction angles as the thickness of the CZTSe/CZTS ratio increases correlates well with the replacement of smaller S atoms with bigger Se atoms. Applying the Vegard law, the anionic compositions [S]/([S] + [Se]) of the bulk CZTSSe thin films were calculated together with the full width at half-maximum (FWHM) values of the (112) reflection for the different CZTSSe absorbers (see Table 1). The results suggest comparable crystalline quality of the synthesized CZTSSe layers compared with that of the pure CZTS and CZTSe reference samples. The AES measurements are presented in Figure 4, and the profiles are almost linear for all of the absorbers, confirming a very high in-depth compositional homogeneity from the surface toward the back interface, where Mo(S,Se)_2_ is formed and an increase of the S content is detected. However, in this region, S was overestimated due to the strong overlapping of the main Mo and S AES emission peaks. For this reason, the composition was evaluated in three distinct regions, front surface, bulk, and back interface, and the values obtained are shown in Table 1. For comparison, the composition values estimated by XRF are also included in Table 1. It can be observed that the different characterization techniques show very similar values of the anionic ratio.

The thickness and morphology of the CZTSSe absorbers were observed by SEM cross-sectional study and compared with those of reference pure selenide CZTSe kesterite. Figure S2 shows that in all the cases, with the introduction of sulfur, a similar morphology with a compact layer is created with thicknesses comprised between 1.6 and 1.7 μm. However, in most of the cases, larger grain sizes in the top region and lower sizes at the bottom were also seen, which was previously mentioned as frequently detected in the kesterite-based absorber layers’ culprit that limits the device performance.^10,12,30^ In our case, this apparent bilayer morphology is however found unrelated to compositional segregation and is thus more likely to be inherent to the surface state of the substrate and its subsequent influence on the film’s growth as previously reported.^31^ Comparing with the CZTSe reference sample, the sequential annealing process proposed here leads to CZTSSe layers with similar bilayer morphology structures but with slightly smaller grains. It is interesting to point out that the Mo(S,Se)_2_ formed at the back contact presents a similar thickness (<200 nm) in all of the cases.

We want also to point out that the processes were reproduced twice for the Sn kesterite with a total of two operators, and in both cases, the results (i.e., the anionic compositional ratios, see Table S1) were almost identical, with very good reproducibility.

### 3.2 Ge Kesterite

**Figure 5:**
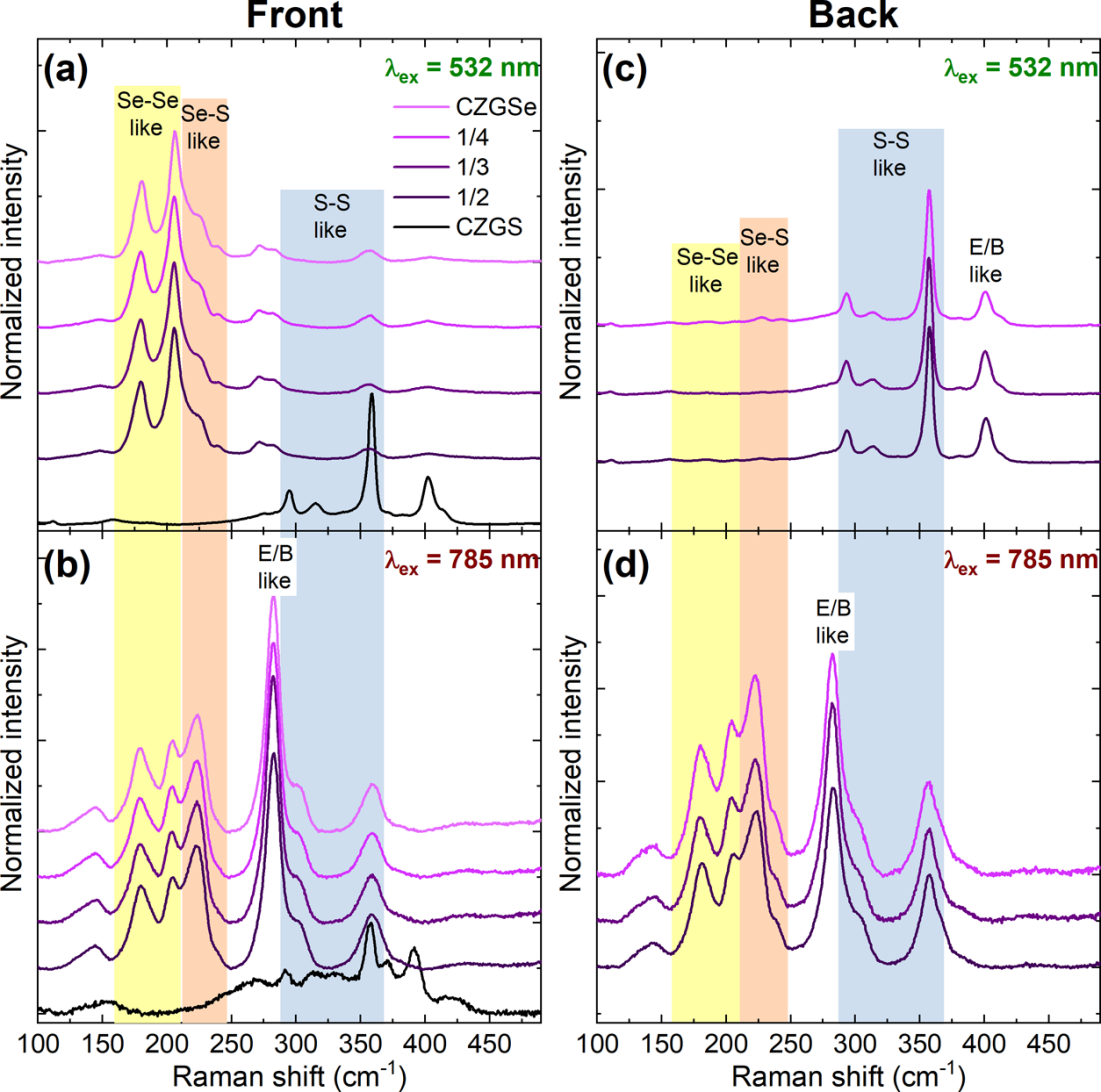
Raman spectra measured at the front of the samples using (a) 532 nm and (b) 785 nm excitation wavelengths for surface and subsurface analysis, respectively, for the different CZGSSe absorbers prepared. Raman spectra of pure CZGSe and CZGS used as references during selenization and sulfurization steps are also shown. Raman spectra measured at the back of the samples (after lift-off) using (c) 532 nm and (d) 785 nm excitation wavelengths, for surface and subsurface analysis, respectively, for the different CZGSSe absorbers prepared.

The first set of CZGSSe samples (labeled as batch 1) was processed using the same processing conditions as those employed for CZTSSe. In this case, and similarly to CZTSSe, the anionic composition of the absorbers was first studied using multiwavelength Raman analysis at the front, i.e., at the surface (532 nm) and subsurface (785 nm), taking advantage of the different penetration depths of the different excitations employed. Similar to the observations made for the CZTSSe solid solution samples, Se–Se-, Se–S-, and S–S-like vibrational modes can be also differentiated in the spectra obtained for the CZGSSe samples. The spectral areas corresponding mainly to these different types of vibrations are highlighted in Figure 5 (in yellow, red, and blue, respectively).

The Raman spectra measured at the front side of the different CZGSSe absorbers are shown in Figure 5a,b, respectively, for 532 and 785 nm excitation wavelengths. Additionally, the Raman spectra of reference samples, corresponding to pure CZGS (initial bottom part of the absorber) and pure CZGSe (added top part of absorber), are also shown in Figure 5 for comparison. The CZGSe reference sample shows small contamination with S, which could come either from the back side of the 1/2, 1/3, and 1/4 samples or is related to accumulated S in the graphite box employed for annealing. The latter is less probable since the box was cleaned prior to annealing; thus, it is believed that S loss is occurring during selenization of the samples. Under 532 nm, all of the CZGSSe samples clearly showed an almost pure CZGSe composition on the surface, similar to the CZGSe reference sample. The [S]/([S] + [Se]) ratio was found to slightly vary from sample to sample but without any correlation with the thickness ratio of precursors. Under 785 nm excitation, the intensity of the E/B-like symmetry peaks was observed to increase significantly. This is explained by the resonance effect of the excitation wavelength employed with the analyzed compound, and this reveals that in the subsurface region, the anionic composition is rather Se-rich. Nevertheless, the slight increase of the S–S-like peaks also observed in these spectra suggests that the S content is higher in the subsurface region than on the very surface (Figure 5a). The [S]/([S] + [Se]) ratio slightly changes from sample to sample but without correlating with the thicknesses ratio of precursors. Combining the measurements performed, it is possible to confirm that all CZGSSe samples present a Se-rich composition on the front surface.

The same analysis was applied to the back side of the layers (after lift-off, see Section 2), and the measured Raman spectra are shown in Figure 5c,d, respectively, for 532 and 785 nm excitation wavelengths. Under 532 nm, the spectra of all of the CZGSSe samples present a high S-rich composition, with an insignificant amount of Se. On the contrary, the spectra measured under 785 nm show a clear resonance effect (also confirmed by the high intensity of the E/B-like peaks), indicating that the laser penetration depth is high enough to pass through the entire S-rich region, which is supposed to be close to half of the film depth (at least for the sample 1/2 and is expected to be less for the sample 1/4).

**Figure 6:**
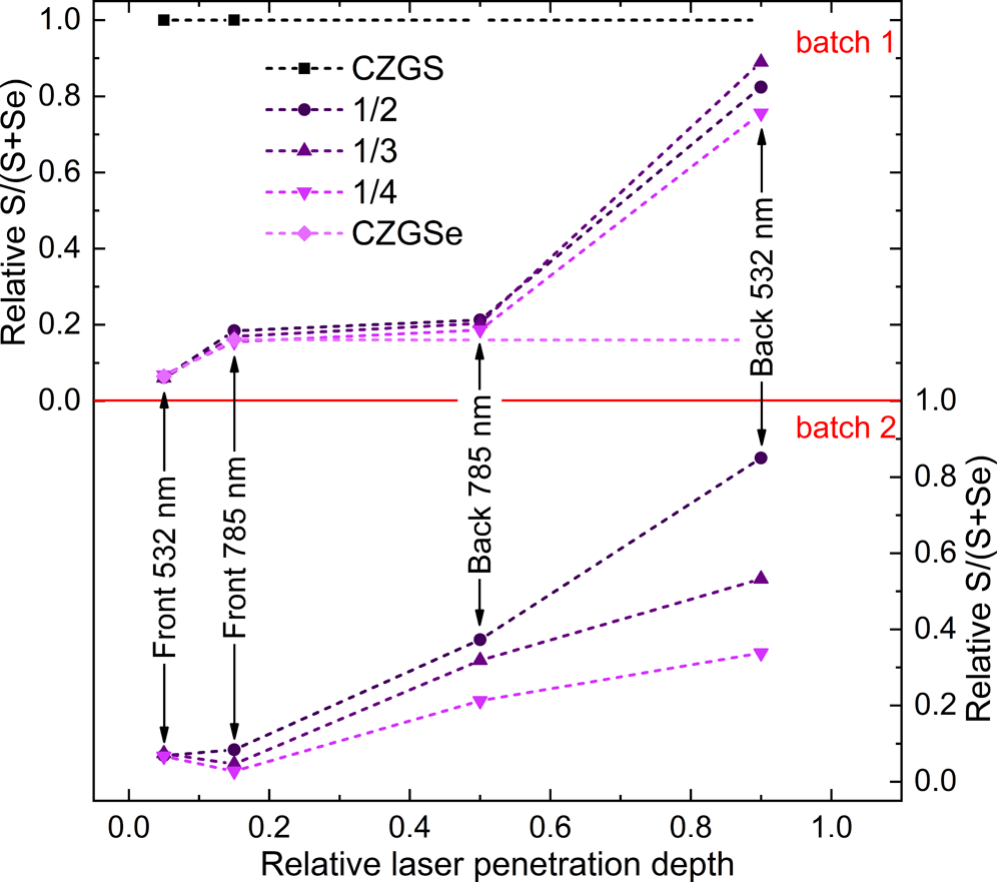
Rough estimation of the relative [S]/([S] + [Se]) ratio as a function of relative laser penetration depth for the CZGSSe absorbers prepared in batch 1 (top) and batch 2 (bottom).

As a result of Raman scattering analysis, it can be concluded a high Se content at the front and high S content at the back side of the CZGSSe thin films, confirming the presence of an in-depth compositional gradient profile. Note that no inhomogeneity from point-to-point measurements in the samples was observed, and no apparent secondary phases were detected using the 532 and 785 nm lasers. Using the Raman scattering spectra discussed above and supposing a linear change of the areas of peaks related to S–S and Se–Se vibrations, a very rough estimation of the [S]/([S] + [Se]) profile was performed (top panel of Figure 6). Although AES would be more reliable to have precise anionic profiling along the absorber thickness, the method here reported is completely valid to identify unambiguously a grading profile and has the main advantage of being faster. However, this could be a limitation for further future optimization of the grading profiles.

Previous investigations on pure CZGSe compounds revealed that slightly different processing conditions (mainly lower selenization temperature) compared to pure CZTSe compounds were needed to get better CZGSe-based devices.^13,25,32^ Taking this into account, the second set of CZGSSe samples (labeled as batch 2) was prepared by lowering the annealing temperatures during selenization (see Section 2).

The same Raman multiwavelength characterization using the lift-off procedure was employed, and the resulting relative [S]/([S] + [Se]) ratio as a function of the relative laser penetration depth is shown in the bottom panel of Figure 6. Similar to the previous batch, at the front side, all of the samples exhibited a Se-rich composition, with insignificant change in the subsurface spectra excited with a 785 nm laser. In contrast to this, spectra measured at the back side of the sample clearly show an enrichment of the Se content with the thickness of the top precursor. As a result, a less abrupt gradient of the anion ratio can be observed for all samples. This indicates the possibility of more precise control of the in-depth gradient by changing the thicknesses ratio of the precursors under optimal selenization temperatures.

All of the results for the fabricated device characterization are shown in the Supporting Information (Figures S3 and S4), and unfortunately, the PV performance was not improved without additional optimizations. Demonstrating that the proposed method will allow improving the PV performance would imply optimizing not only the absorber band gap profile but also the different interfaces in contact with the absorber, which is beyond the scope of the proposed work. Nevertheless, a discussion supported with simulation results is also included to explain the observed device behavior.

## 4 Discussion

**Figure 7:**
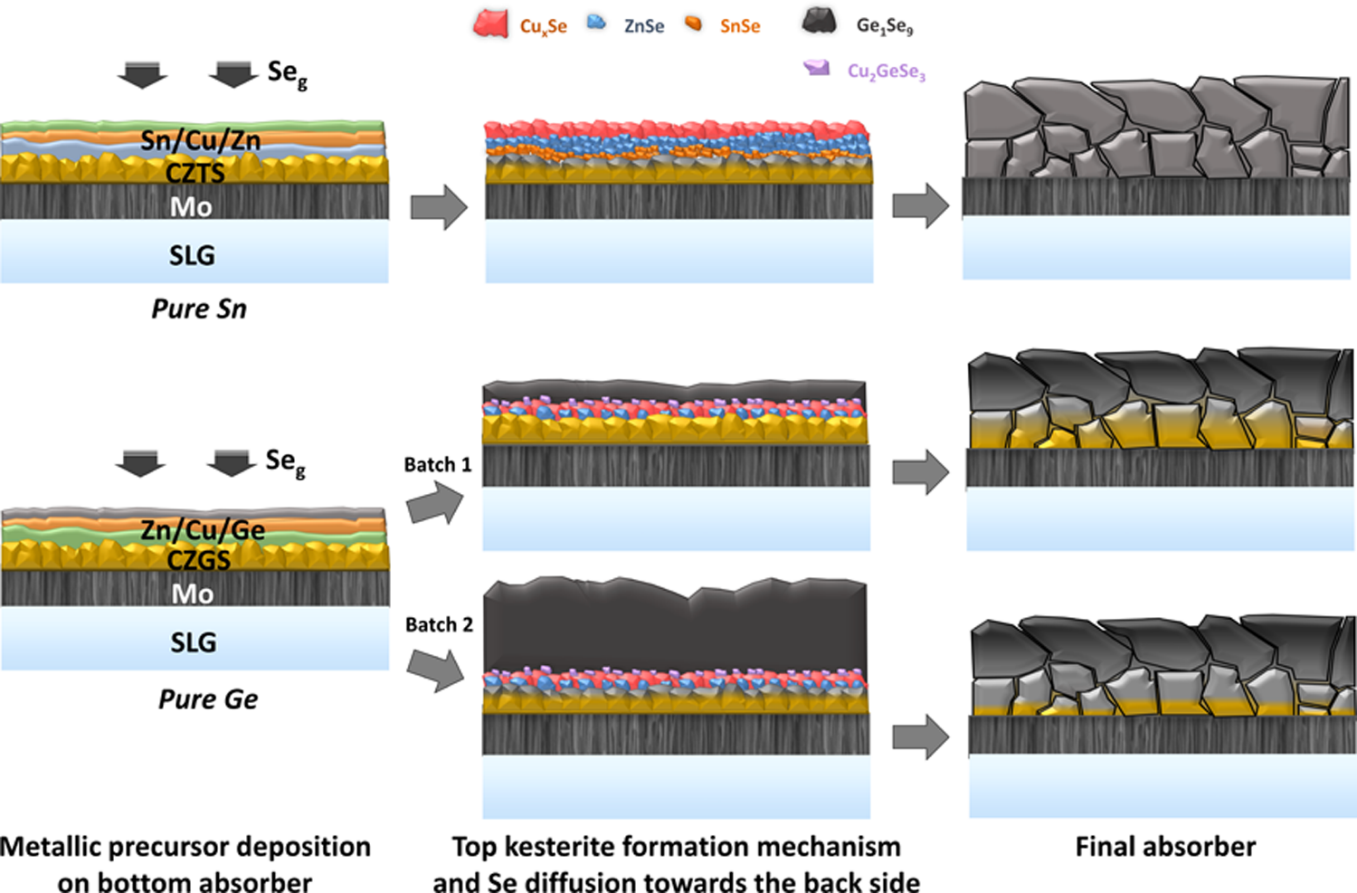
Schematic sketches of different intermediate steps and top kesterite reaction mechanism and Se diffusion involved in the formation of the solid solution absorbers prepared in this work.

Following a similar experimental process between samples, it has been shown that a homogeneous anionic composition is achieved for CZTSSe, while a graded anionic composition is obtained with CZGSSe. The anionic composition for CZTSSe is perfectly controlled with the thickness ratio of the sulfide and selenide absorber parts, varying from approximatively 45 to 20%. For CZGSSe, the composition varies from an almost pure selenide part (approximatively 10% of the relative value) at the front to an almost sulfide part (approximatively 80% of the relative value) at the back for certain processing conditions (batch 1) and with an increased intermixing (comprising approximatively between 40 and 80% of the relative values, depending on the sulfide part thickness) by adjusting the processing parameters (batch 2). This result shows the potential of the technique and that the anionic composition can be finely tuned in a homogeneous or graded form within the absorber thickness. The elemental interplay responsible for the different S–Se dynamics between compounds is not yet completely understood and is beyond the scope of this study, which aims at reporting the end result and potential of the method. Nevertheless, several possible hypotheses can be made. During selenization, two reactions are involved sequentially or at the same time: (1) selenization of the top metallic precursors and (2) anion substitution. Regarding reaction (1), a difference in the formation mechanism of Ge and Sn kesterite has already been reported elsewhere.^7,25^ In the case of the formation of CZGSe, the presence of a Se-rich Ge–Se liquid phase during the initial moment of annealing acts as a Se reservoir and is key to controlling Se incorporation. During the formation of the CZGSe phase, Se diffusion occurs via this thick eutectic liquid phase formed on the surface,^25^ while for CZTSe, Se diffusion is mainly controlled from a thin compact Cu*_x_*Se phase layer on the surface.^7^ At 400 °C, this Ge–Se liquid phase has probably been largely reduced in thickness (i.e., Se being evaporated) and thus less Se is available to diffuse. This explains why for batch 1, a low amount of Se is available for substitution of S (reaction 2) in the bottom CZGS part of the absorber, inducing, thus, a strong anionic gradient. By reducing the temperature to 380 °C in batch 2 for the formation of CZGSe, a thicker liquid phase remains on the surface and thus more Se is available to diffuse and substitute S (reaction 2 is promoted) in the CZGS bottom laye, inducing less abrupt gradient. However, for the case of CZTSSe, reaction 2 is promoted, leading to the formation of a homogeneous composition within the thickness. Other mechanisms could be involved to explain this observation and especially a possible degradation of the bottom sulfide part into binary phases. This could result in an intermixing of different sulfide and selenide phases, leading to the formation of a kesterite layer with a fix solid solution composition. It could even be hypothesized that this degradation could be higher for the bottom Sn sulfide part than the Ge one, as degradation of the CZTS/Mo interface is a well-known problem.^33^ The metal in contact with the sulfide part (i.e., Sn in the case of CZTSe and Zn in the case of CZGSe) has maybe also some role to play. For example, Sn could be reacting with the bottom part, creating volatile Sn*_x_*(S,Se)*_y_* phases that react with the top and bottom parts, leading faster to homogeneity. In any case, these differences obtained between Ge and Sn kesterite clearly reveal a different Se incorporation dynamics, which seems to be highly dependent on the involved cations and also probably on the stacking order of the metal precursor. In a similar way, it was recently reported that depending on the stacking order, the formation mechanism of Ge-based kesterite proceeds at different speeds, and putting Ge on top delays the formation reaction.^34^ Finally, Figure 7 depicts the schematic representation summarizing the proposed mechanisms involved in the formation of Ge- and Sn-based kesterite in this work.

## 5 Conclusions

In this paper, an innovative method for the formation of kesterite absorbers with very well controlled anionic compositions is reported. A simple technique consisting of first preparing a pure sulfide absorber at the bottom and completing it with a metallic precursor to be selenized on the top allows us to obtain fixed (constant) or graded compositions in Sn- and Ge-containing kesterites. It is shown that this depends mainly on the different kesterite formation mechanisms, imposed by the top metallic stacking order, and on the processing parameters used for selenization, as a way of regulating the Se incorporation and diffusion toward the bottom pure sulfide part and avoiding/promoting substitution. These results demonstrate the potential of this simple technique and could pave the way to define and standardize anionic compositional profiles not only for kesterite but also for other chalcogenide-related technologies.

## Supporting Information Available

Raman spectra of CZTSSe absorbers on the surface and cross-sectional SEM images of CZTSe reference samples (PDF)
