# Supplementary material for: Controlling the Anionic Ratio and Gradient in Kesterite Technology
Source: ACS Appl Mater Interfaces. 2022 Jan 3;14(1):1177–86. doi: 10.1021/acsami.1c21507 (PMC8762644; doi:10.1021/acsami.1c21507)
Supplement: Supplementary file 1 — am1c21507_si_001.pdf [file am1c21507_si_001.pdf]

Supporting Information

Controlling the anionic ratio and gradient in kesterite technology

Jacob Andrade-Arvizu,[†,∥] Robert Fonoll Rubio,[†,∥] Victor Izquierdo-Roca,[†] Ignacio Becerril-Romero,[†] Diouldé Sylla,[†] Pedro Vidal-Fuentes,[†] Zacharie Jehl Li-Kao,[‡] Angélica Thomere,[†] Sergio Giraldo,[†] Kunal Tiwari,[†] Shahaboddin Resalati,[§] Maxim Guc,[†] and Marcel Placidi*[†,‡]*

[†]Solar Energy Materials and Systems (SEMS), Institut de Recerca en Energia de Catalunya (IREC), Jardins de les Dones de Negre 1, 08930 Sant Adrià de Besòs, Barcelona, Spain
[‡]Departament d'Enginyeria Electrònica, Universitat Politècnica de Catalunya, C/ Jordi Girona 1, 08034 Barcelona, Spain
[§]Architectural Engineering Research Group, Oxford Brookes University, United Kingdom

Corresponding Authors

*mguc@irec.cat, *marcel.placidi@upc.edu

Author Contributions

The manuscript was written through contributions of all authors. All authors have given approval to the final version of the manuscript. ∥ These authors contributed equally to this work.

Raman spectra and SEM images of Sn kesterite samples

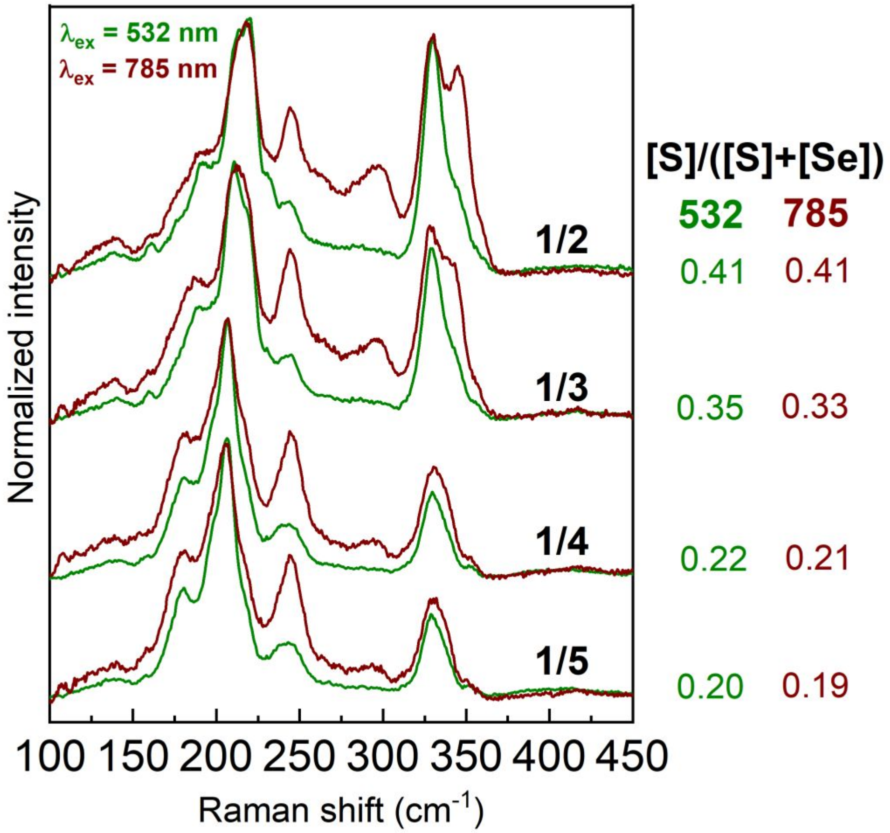

Figure S1. Raman spectra of the CZTSSe absorbers at the surface (using 532 nm excitation wavelength) and subsurface (using 785 nm excitation wavelength); in the right part, the anionic compositional ratio is indicated, for both excitation wavelengths.

Table S1. Anionic compositional ratios for different batches of CZTSSe absorbers (estimated from Raman spectra of the absorbers surface, using 532 nm excitation wavelength).

	**[S]/([S]+[Se])**	
**Samples**	**Batch 1**	**Batch 2**

**1/2**	0.41	0.41
**1/3**	0.35	0.31
**1/4**	0.22	0.19
**1/5**	0.20	0.17

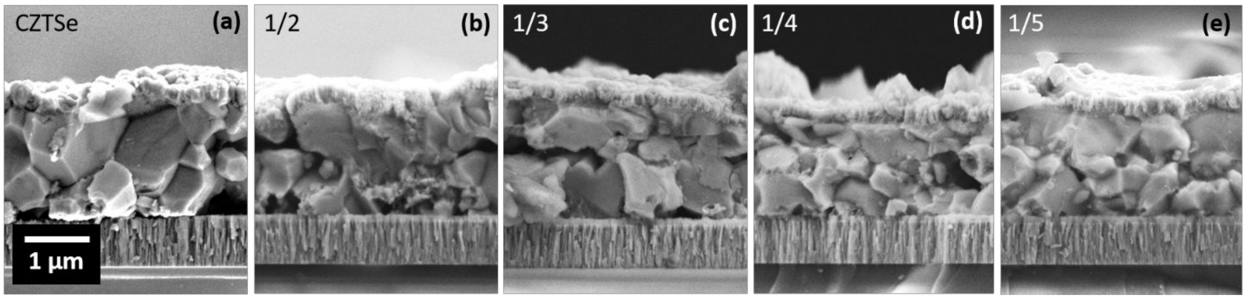

Figure S2. Cross-section SEM images of (a) a CZTSe reference samples, and (b)(c)(d)(e) the different CZTSSe prepared absorbers with different thicknesses of the bottom sulfide layer.

Devices results, simulation and discussion

The JV characteristics for the Sn kesterite solar cells with different composition ratios are shown in Fig. S3 below. In contrast to what was expected, the V_{oc} was not increasing with higher anionic composition. It has been reported, even recently, that the introduction of S in Kesterite leads to the formation of an interface defect which may limit the voltage.[1] Indeed, SCAPS modelling was performed starting from a baseline Kesterite example, and introducing a fermi level pinning from single acceptor defects at the front interface and related to the presence of S. As shown in Figure S3, the pinning of the fermi level (right side) reproduces accurately the experimental behavior of the devices. Hence, it is likely that the voltage limitation occurs due to the presence of such donor defects at the absorber/buffer interface.

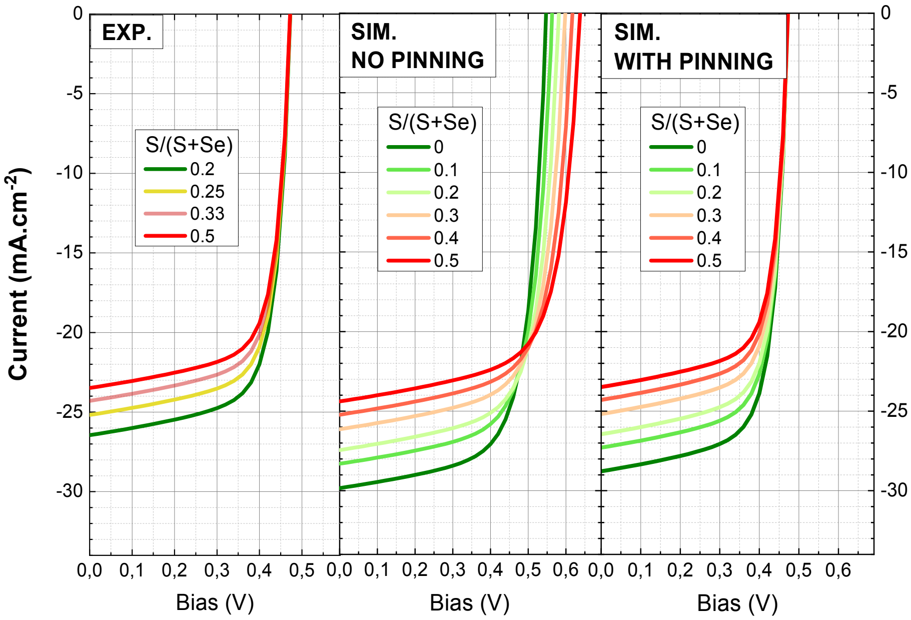

Figure S3. Current-voltage characteristics of the Sn kesterite devices made with different anionic ratios: (left) experimental results, and SCAPS simulation without (center) and with (right) fermi level pinning.

The JV characteristics of the Ge kesterite solar cells with the different compositional profiles are shown in Fig. S4. In this case, only the devices from batch 1 are represented, as the ones from batch 2 were not working. We believe the main reason is due to the higher S content towards the center of the absorber, that could result in a higher defective bulk, as the deep defect cluster Ge_{zn}+Cu_{Zn} becomes predominant and highly detrimental with a concentration exceeding the absorber's carrier concentration.[2]

Once again, SCAPS modelling considering the simultaneous presence of interfacial defects, and the existence of literature reported defect clusters shows a similar behavior akin to that of batch 1. Indeed, while the V_{oc} is marginally modified (and in ways that make us believe that different competing factors may exist), a strong variation of the current is observed. While it is easy to understand it in terms of bandgap modification, as high S content samples have expectedly a lower current, it is also worth mentioning that the introduction of S in Kesterite leads to the prevalence of a cluster defect with a higher carrier capture cross section ($\sigma_{p,n}$); indeed, while the Ge_{zn}+Cu_{Zn} cluster has a reported $\sigma_{p,n}$ = 10^{-14}, it is reported as 10^{-14} for a pure S content. Such difference may also contribute to reducing the current, as the defect interact more with electrons and holes.

The SCAPS modelling including state of the art defects shown Figure S4 goes in the same direction, although those curves aren´t fully quantitative as the real bulk and interface defect density was not measured in our cells, and the data from the supplementary information of reference have been used instead.[2]

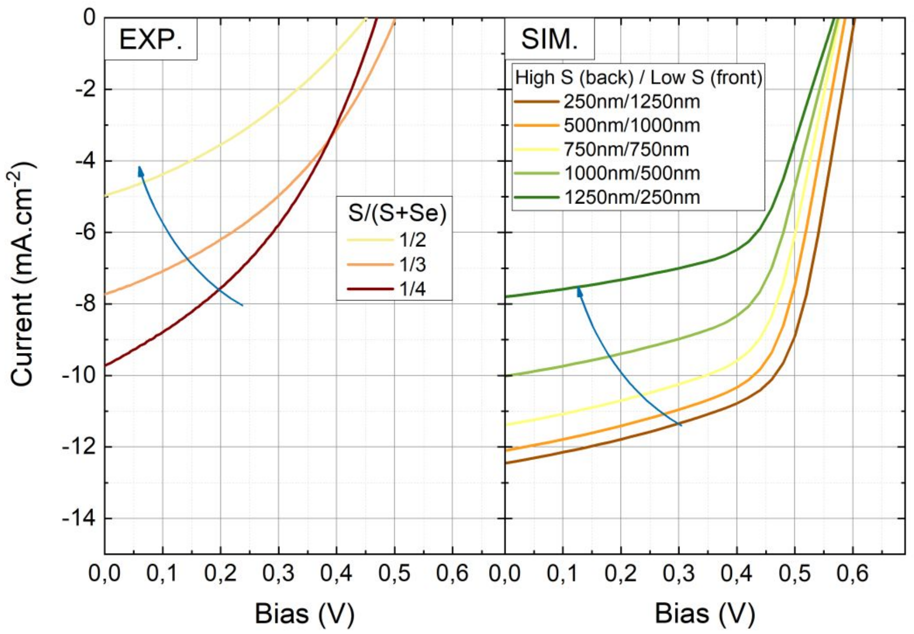

Figure S4. Current-voltage characteristics of the Ge kesterite devices made with different graded anionic profiles: (left) experimental results, and (right) SCAPS simulation including interfacial and cluster defects (here, the high S content thickness of the back side was gradually varied)

References

1. Li, J.; Huang, J.; Huang, Y.; Tampo, H.; Sakurai, T.; Chen, C.; Sun, K.; Yan, C.; Cui, X.; Mai, Y.; Hao, X., Interface Recombination of Cu2ZnSnS4 Solar Cells Leveraged by High Carrier Density and Interface Defects, Solar RRL **2021**, 5, 10, 2100418.

2. Kim, S.; Marquez, J.A.; Unold, T.; Walsh, A., Upper limit to the photovoltaic efficiency of imperfect crystals from first principles, Energy Environ. Sci. **2020**, 13, 1481-1491.
